# The Role of Histone Acetylation and the Microbiome in Phytochemical Efficacy for Cardiovascular Diseases

**DOI:** 10.3390/ijms21114006

**Published:** 2020-06-03

**Authors:** Levi W. Evans, Maheshi Athukorala, Kristina Martinez-Guryn, Bradley S. Ferguson

**Affiliations:** 1 Department of Nutrition, University of Nevada Reno, Reno, NV 89557, USA; levi659@gmail.com (L.W.E.); maheshikathukorala@nevada.unr.edu (M.A.); 2 Department of Biomedical Sciences, Midwestern University, Downers Grove, IL 60515, USA; 3 Center of Biomedical Research Excellence for Molecular and Cellular Signal Transduction in the Cardiovascular System, University of Nevada Reno, Reno, NV 89557, USA

**Keywords:** phytochemicals, histone acetylation, histone deacetylase (HDAC), microbiome, microbiota, heart

## Abstract

Cardiovascular diseases (CVD) are the main cause of death worldwide and create a substantial financial burden. Emerging studies have begun to focus on epigenetic targets and re-establishing healthy gut microbes as therapeutic options for the treatment and prevention of CVD. Phytochemicals, commonly found in fruits and vegetables, have been shown to exert a protective effect against CVD, though their mechanisms of action remain incompletely understood. Of interest, phytochemicals such as curcumin, resveratrol and epigallocatechin gallate (EGCG) have been shown to regulate both histone acetylation and microbiome re-composition. The purpose of this review is to highlight the microbiome–epigenome axis as a therapeutic target for food bioactives in the prevention and/or treatment of CVD. Specifically, we will discuss studies that highlight how the three phytochemicals above alter histone acetylation leading to global changes in gene expression and CVD protection. Then, we will expand upon these phytochemicals to discuss the impact of phytochemical–microbiome–histone acetylation interaction in CVD.

## 1. Introduction

Cardiovascular diseases (CVD), which include heart attack, atherosclerosis, hypertension, stroke and heart failure, remain a leading public health concern. Indeed, CVD is the leading cause of death; CVD causes 1 of every 3 deaths globally [1]. Additionally, more than one-third of Americans live with some form of CVD, costing approximately $351.2 billion per year [1]. These are alarming statistics given the development of ground-breaking cardiovascular pharmacotherapies in recent decades. The high prevalence and mortality rates are tied to the high financial costs, thus highlighting the need for better and more affordable CVD therapies. With this in mind, investigators have begun to elucidate the role of the epigenome and microbiome in human health and disease in an effort to identify novel therapeutic targets for CVD.

Epigenetic modifications regulate gene expression independently from changes in the DNA sequence and include lysine residue acetylation on histone protein tails [2]. Lysine acetylation is a reversible event and catalyzed by histone acetyltransferase (HAT) enzymes while lysine deacetylation is catalyzed by histone deacetylase (HDAC) enzymes [3]. Histone acetylation is generally associated with relaxation of the chromatin allowing increased gene transcription whereas histone deacetylation is associated with decreased gene transcription. Currently, 18 mammalian HDACs have been identified and divided into four separate classes: class I (HDACs 1, 2, 3 and 8), class II (HDACs 4, 5, 6, 7, 9 and 10), class III (SIRT1-7) and class IV (HDAC11). Class II HDACs are subdivided further into class IIa (HDACs 4, 5, 7 and 9) and IIb (HDACs 6 and 10). Class I, II and IV HDACs require zinc as a cofactor to catalyze deacetylation, while class III HDACs (sirtuins; SIRT1-7) require nicotinamide adenine dinucleotide (NAD+). Relevant to this review, zinc-dependent HDAC inhibition is efficacious in animals models for CVD and has been shown to attenuate cardiac hypertrophy [4], scarring [5] and dysfunction [6]. Conversely, sirtuin hyperactivation seems to be beneficial to the heart [7]. Thus, therapeutics that target HDAC (in)activity in the heart may bridge the gaps of current CVD pharmacotherapies but warrant further investigation.

The human body hosts trillions of microorganisms, with the highest densities found in the gastrointestinal (GI) tract. Indeed, microbial densities in the colon can reach 10^11^ colony-forming units per gram of content (cfu/g) [8]. These observations demonstrate that the gut microbiome is considerably larger than the genome of its host, and not surprisingly, has been heavily investigated for its role in regulating health and disease. Similar to changes in histone acetylation, the gut microbiome has been suggested to play a key role in cardiovascular health and disease. Consistent with this postulate, changes in microbial communities have been linked to CVD-related events and risks [9], including atrial fibrillation [10], heart failure [11] and elevated blood lipids and cholesterol [12,13]. In addition, the gut microbiota has also been shown to modulate HDAC activity in the gut [14] and may serve as a critical bridge between the beneficial effects of polyphenols and improved cardiac function.

The etiology of CVD is multifactorial and includes both unmodifiable and modifiable risk factors. According to the American Heart Association (AHA), aging, gender and heredity are classified as uncontrollable risk factors for CVD. Modifiable risk factors include tobacco use, physical activity and diet. In fact, diet is now considered the leading modifiable risk factor for many diseases including CVD [15], and a substantial portion of cardiovascular diseases can be prevented through dietary modification [16]. For example, the Mediterranean Diet and the DASH (Dietary Approaches to Stop Hypertension) Diets are two commonly recommended dietary interventions that help manage and prevent heart disease [17]. Both heart-healthy diets are abundant in plant-based foods like fruits, vegetables, whole grains, legumes, nuts and seeds. While these foods are nutrient-dense, they are also rich in non-nutritive plant compounds, i.e., phytochemicals, which play important roles in heart health.

Phytochemicals were originally studied for their anti-oxidative and anti-inflammatory potential [18]. However, recent evidence has emerged that these compounds can also regulate histone acetylation and impact microbial growth. In this review, we will discuss the phytochemical–microbiome–epigenome axis, with an emphasis on the regulation of histone acetylation and human health and disease. 

## 2. Phytochemicals, Histone Acetylation and CVD

Phytochemicals are compounds naturally found in plants that protect their hosts against pollution, stress, drought, UV exposure and pathogenic attack [19]. Although these xenobiotic compounds were originally thought to be metabolized and excreted as waste, evidence over the past several decades has shown that phytochemicals can improve human health. Indeed, plants abundant in phytochemicals have been traditionally used to treat bacterial and viral infections as well as gastrointestinal distress [20]. However, a mounting body of literature has implicated natural dietary compounds as players in the regulation of intracellular signaling cascades, inflammation and oxidative stress in many non-communicable diseases including CVD [20]. Concerning heart disease, phytochemicals have been shown to protect against atherosclerosis, thrombosis, ischemia, hyperglycemia, hyperlipidemia and hypertension [21]. While early work focused on cellular signaling, inflammation and oxidative damage, recent evidence shows that phytochemicals serve as epigenetic modifiers to also protect the heart. Below, we will discuss the role of three phytochemicals in the regulation of histone acetylation and CVD.

### 2.1. Curcumin and Histone Acetylation in CVD

Turmeric (*Curcuma longa*) is a plant often used as a spice, food coloring agent, preservative and holistic remedy for treating disease [22]. Curcumin is credited as the primary bioactive compound of turmeric and has been shown to protect the heart [23,24]. With regard to histone acetylation, curcumin has been shown to inhibit p300 histone acetyltransferase (HAT) activity [25]. Activation of p300 contributes to histone acetylation of transcription factors such as GATA binding protein 4 (GATA4), which promotes pathological cardiac remodeling by upregulating pro-hypertrophic, (e.g., atrial natriuretic factor (ANF) and beta myosin heavy chain (β-MHC)), and pro-inflammatory, (e.g., endothelin-1 (ET-1)), gene expression [26]. Indeed, curcumin was shown to attenuate cardiac hypertrophy, fibrosis and dysfunction by inhibiting p300-mediated GATA4 acetylation [25]. The ability of curcumin to inhibit p300 activity was further shown to inhibit pro-inflammatory gene expression, specifically triggering receptors expressed on myeloid cells-1 (TREM-1) [27], that induce atherosclerosis and myocardial infarction [28]. Thus, inhibiting p300 seems to be an important mechansistic action of curcumin for its cardioprotective role.

Curcumin has also been shown to attenuate cardiac remodeling by inhibiting zinc-dependent HDAC activity [29]. For example, curcumin reduced HDAC1 binding and increased histone acetylation at the promoter region of tissue inhibitor of metalloproteinase 1 (TIMP1), which subsequently resulted in increased TIMP1 expression and reduced fibrosis in spontaneously hypertensive rat hearts [30]. Curcumin was also shown to suppress the antagonistic target of TIMP1, matrix metalloproteinase-2 (MMP-2) [30]; MMP2 promotes cardiac fibrosis and dysfunction [31,32]. Others have confirmed that curcumin is anti-fibrotic by targeting TIMP/MMP2 expression [33]. Not surprisingly then, hypertensive rats treated with curcumin had improved fractional shortening (i.e., contractile function) and cardiac fibrosis in addition to attenuated wall thickening and myocardial cell enlargement (i.e., cardiac hypertrophy) compared to hypertensive nontreated rats [30]. While it is counterintuitive that curcumin inhibits both HATs and HDACs in a manner that protects the heart, it is likely that HATs and HDACs regulate different histone and transcription factor targets in the myocardium.

#### Translational Role for Curcumin in CVD

In agreement with the cardioprotective actions in experimental models characterized above, evidence also supports cardioprotection for curcumin in humans. Studies have reported that curcuminoids, at doses ranging from 20 mg/day to as high as 1500 mg/day and treatment periods ranging from 2 weeks to as long as 6 months, reduced circulating markers associated with atherosclerotic events, including total cholesterol (TC), low-density lipoprotein cholesterol (LDL), triglycerides (TG), lipoprotein A (Lp(a)) and apolipoprotein B (Apo B) [24,34,35,36]. Additionally, 1500 mg/day of a curcuminoid extract given for 24 weeks improved pulse wave velocity (PWV), a marker associated with arterial disease, as well as improvements in metabolic markers, including adiponectin and leptin expression, that coincided with improved insulin resistance in male and female diabetics [24]. Similarly, 4000 mg/day of curcuminoid supplementation 3 days prior to and 5 days post coronary artery bypass grafting (CABG) was shown to reduce post-operative oxidative stress (malondialdehyde: MDA), inflammation (C-reactive protein: CRP) and myocardial infarction compared to a placebo control [37]. Lastly, pre-surgical curcumin supplementation at 45 mg/day for 14 days reduced c-Jun N-terminal kinase (JNK) activity and apoptotic caspase-3 expression in cardiomyocyte nuclei from biopsied heart tissue of 2–6-year-old children who underwent tetralogy of Fallot surgery [38]. Post-operative cardiac function was also improved in the curcumin-supplemented group [38]. Data from Sukardi et al. [38] are particularly interesting, as: (1) oral administration of curcumin affected intracellular signaling cascades in the human heart, previously shown to be regulated by the class I HDACs in cardiac myocytes [39], and (2) the effective oral dose of curcumin used in this human study (45 mg/day or 3.7 mg/kg/day) [38] was lower than the effective dose (50 mg/kg/day) that ameliorated heart failure in spontaneously hypertensive rats [30]. While scientists are currently developing curcumin derivatives that have bioactive properties in humans reflective of those in rodents [40], the above data suggest that curcumin is a viable therapeutic strategy for select CVDs.

### 2.2. Resveratrol and Histone Acetylation in CVD

Resveratrol is a stilbene naturally found in peanuts, grapes, red wine and some berries [41]. Many studies have shown that resveratrol activates NAD+-dependent class III HDACs, the sirtuins, contributing to improved CVD outcomes in animal models [42]. Indeed, resveratrol has been shown to attenuate cardiac hypertrophy, fibrosis and dysfunction and increase sirtuin activity and intracellular calcium handling [43]. Several of these studies have linked resveratrol to sirtuin-mediated deacetylation and cardiac function. For instance, Sin et al. showed that resveratrol-induced sirtuin 1 activation deacetylated forkhead box protein O1 (Foxo1) and reduced expression of the downstream apoptotic signaling molecule of Foxo1, Bcl-2 interacting mediator of cell death (Bim), in aged mouse hearts [44]. Cardiomyocyte apoptosis results in cardiac dysfunction and, eventually, heart failure [45]. In agreement with these data, aged mice treated with resveratrol had reduced cardiomyocyte death and improved cardiac function compared to controls [44]. Further reports have confirmed that resveratrol-induced sirtuin 1 activity protected cardiomyocytes from Foxo1-mediated apoptosis [46]. In addition to its anti-apoptotic role, resveratrol-mediated sirtuin 1 activation deacetylated endothelial nitric oxide synthase (eNOS), which led to increased eNOS activity and nitric oxide (NO) production in endothelial cells [47]. NO regulates endothelial function and reduces oxidative stress in the vasculature [48]. Consistently, resveratrol concomitantly upregulated eNOS expression, improved endothelial dysfunction and attenuated plaque formation in an atherosclerotic mouse model [49]. Thus, resveratrol can increase sirtuin activity in order to protect against CVD. 

To our knowledge, no study has linked the cardioprotective actions of resveratrol with zinc-dependent HDAC inhibition. However, resveratrol has been shown to inhibit non-sitruin HDAC activity in cancer models [50]. Additionally, resveratrol has been shown to regulate the pro-inflammatory pathways, nuclear factor kappa B (NF-κB) [51] and mitogen-activated protein kinases (MAPKs) [52], which have been implicated with zinc-dependent HDACs in the heart [39,53]. While the current state of the literature suggests that the primary action of resveratrol is regulating sirtuin activity, further investigation examining the role of zinc-dependent HDACs in the heart-healthy actions of resveratrol is warranted. 

#### Translational Role for Resveratrol in CVDs

Similar to curcumin, human studies suggest that resveratrol is a promising bioactive food compound that protects the heart. Two meta-analyses concluded that resveratrol at doses between 150 mg/day and 1000 mg/day reduced systolic blood pressure [54,55]. In addition to its anti-hypertensive actions, 500 mg/day (approximately 6 mg/kg/day) of resveratrol supplemented for 30 days improved circulating cholesterol levels and oxidative stress concomitant with upregulated Sirt1 and peroxisome proliferator-activated receptor-gamma (PPARγ) expression in peripheral blood mononuclear cells (PBMCs) in type 2 diabetic patients with coronary artery disease [56]. These data are interesting as resveratrol was shown to regulate catalytic enzymes of histone acetylation in humans [56] at doses similar to those used in experimental models described above at 4.9 mg/kg/day [44] and by others at 5.2–22.4 mg/kg/day [57]. Similar to these findings, a resveratrol-enriched (4–80 mg/g) grape extract supplemented for six months lowered the pro-atherosclerotic molecules and oxidized LDL (LDLox) and apolipoprotein B (ApoB) more so than a non-resveratrol-enriched grape extract or a placebo control in primary cardiovascular disease prevention (PCP) patients [58]. While these studies suggest resveratrol imparts heart health through Sirt1-mediated mechanisms, other human studies have shown that resveratrol has no such effect on Sirt1 or clinical markers relevant to CVD [59]. These confounding results in human studies may be because resveratrol was shown to be less effective at lower doses [54,55]. While questions concerning resveratrol dosing or lower levels of NAD+ in diseased patients (NAD is needed for sirtuin activity) need to be further clarified, many human findings support resveratrol as a potential therapuetic for CVD. 

### 2.3. EGCG and Histone Acetylation in CVD

Green tea is a beverage constantly characterized as heart-healthy due to its rich flavonoid content, particularly the flavonoid epigallocatechin-3-gallate (EGCG) [60]. Indeed, reports have elucidated several efficacious actions of EGCG in the heart [61], with recent findings linking the cardioprotective actions of EGCG to histone acetylation. In one report, EGCG reversed hypoacetylation at the proximal promoter region of cardiac troponin I (cTnI) by reducing HDAC1 expression in aging mice [62]. cTnI is a critical driver of diastolic function, and its expression is regulated by cTnI promoter acetylation [63]. Not surprisingly, then, EGCG increased cTnI expression and improved cardiac diastolic function [62]. EGCG was also shown to inhibit HDAC1 activity and increase histone acetylation of the sarcoplasmic reticulum Ca-ATPase 2a (SERCA2a) promoter in transverse aortic constricted (TAC) mice [64]. SERCA2a is a critical regulator of calcium handling during cardiac muscle contraction. Loss of SERCA2a contributes to heart dysfunction [65]. Consistent with this, EGCG attenuated cardiac hypertrophy, fibrosis and dysfunction in TAC-induced mice [64]. Taken together, EGCG elicits protective effects in the heart, in part, through HDAC inhibition that promotes increased histone acetylation and gene expression of proteins critical for contraction and relaxation.

In addition to its role in the myocardium, EGCG has been shown to attenuate vascular inflammation via changes in histone acetylation [66]. Here, EGCG reduced binding of NF-κB subunit p65 and HAT p300 but increased binding of HDAC1 and HDAC2 at the promoter regions of NF-κB-regulated genes in PCB-126 endothelial cells [66]. These data were linked with hypoacetylation at promoter regions and suppression of inflammatory and atherosclerotic gene expression, which suggests that EGCG prevents inflammation-induced atherosclerosis by regulating histone acetylation [66]. The authors from this study note that EGCG doses were substantially higher than those observed in humans. 

#### Translational Role for EGCG in CVDs

Translational findings reported that brachial artery flow-mediated dilation (FMD), a marker of endothelial function that predicts heart disease [67], improved in patients with coronary artery disease two hours after EGCG supplementation at 300 mg [68]. In contrast, EGCG supplementation of 300 mg/day for 14 days did not improve FMD in these patients, likely due to EGCG being depleted in the plasma after the two-week treatment period [68]. Notably, FMD of healthy men did not improve two hours after a single dose of EGCG at 200 mg [69]. In relation to hypertension, systolic blood pressure was shown to improve after 14 days of consuming 250 mL/day, roughly 200 mg/day of EGCG, of green tea [70]. In contrast, others reported that 300 mg/day of EGCG supplementation for two days or two weeks did not improve systolic or diastolic blood pressure [68]. These data suggest that EGCG supplementation at doses greater than 200 mg for acute periods of time, but not for periods longer than two weeks, benefit endothelial function independently from hypertensive indices; whether this confers cardioprotection remains unclear. However, the contradictory findings concerning EGCG efficacy in humans may also be due to low concentration doses. For example, doses of EGCG used in experimental animal and cell models approximated 50 mg/kg/day [62,64] or 15–30 μM [66], which were higher than the majority of human studies discussed. One favorable human report did observe EGCG efficacy at doses of 50 mg/kg/day for 12 months [71]. Future studies that examine dose-and-time relationships would improve our understanding of how much and for how long one should consume EGCG to benefit the heart. 

Aside from the above reports [68,69], few human studies have delineated the lone role of EGCG in CVD prevention. However, the effects of EGCG-rich teas have been extensively studied. In relation to atherosclerotic risks, oxidative indices which were measured by plasma thiobarbituric-acid reactive substances (TBARS), trolox equivalent antioxidant capacity, superoxide dismutase, catalase, total glutathione, glutathione peroxidase and glutathione reductase, as well as ox-LDL lagtime, concomitantly improved in patients with mild hypercholesterolemia who consumed either an EGCG-rich green tea or EGCG-rich oolong tea for 12 weeks, with EGCG doses of 228.4 mg/day or 192.2 mg/day, respectively [72]. Additionally, EGCG-rich teas, at doses as low as 2.5 mg/kg/day [72] and as high as 50 mg/kg/day for 12 months have been shown to reduce LDL cholesterol [71]. Finally, two observational reports [73,74] suggests that consuming EGCG-rich green teas, with EGCG doses between 500–800 mg/day and plasma concentrations between 500 and 600 nM, for 12 months attenuated transthyretin amyloidosis-induced cardiac remodeling (e.g., left ventricle wall thickness and mass) and dysfunction (e.g., ejection fraction). EGCG was not the principal bioactive compound in teas shown to reduce CVD risk markers characterized above [69]. As such, the beneficial effects of green tea may be independent of EGCG supplementation. 

## 3. Microbiome

As mentioned above, the human body hosts trillions of microorganisms, which reside primarily on epithelial surfaces and gut lumen and attain their highest densities within the GI tract. Here, the density of microbiota populations varies along the small intestine, cecum and large intestine (colon) as each GI region houses distinct microbial habitats that are affected by chemical and nutrient gradients. Specifically, the small intestine is more acidic and contains more antimicrobial compounds, which results in reduced microbial densities [75]. Moving down the GI tract, pH and, with it, microbial densities increase. In fact, microbial densities in the colon reach 10^11^ colony-forming units per gram of content (cfu/g) compared to 10^2^ cfu/g in the small intestine [8]. 

The human gut consists of a variety of bacteria, mainly species that belong to *Bacteroidetes*, *Firmicutes, Actinobacteria, Proteobacteria* and *Cerrucomicrobia phylum.* Among them, *Bacteroidetes* and *Firmicutes* are the most prominent species [76] and are often used as a ratio to mark pathological gut dysbiosis [77]. Gut microbiota regulate, in part, host immunity, energy metabolism, hormonal balance and fermentation and synthesis of metabolites such as short-chain fatty acids (SCFA), amino acids and vitamins [8]. The composition of the gut microbiome of a healthy person can vary considerably from one person to another. This variation between individuals is due to a number of factors including age [78], environment [79], prescription drug use [80] and, of interest, diet [81]. As the gut microbiome is considerably larger than the genome of its host, a shift in focus has emerged towards understanding and targeting the microbiome in health and disease. 

### Microbiome and CVD

A growing body of evidence suggests gut microbiota and its metabolites are involved in cardiovascular health and disease. For example, Jie et al. found distinct differences in microbial taxa between 187 healthy controls and 218 patients diagnosed with atherosclerotic cardiovascular disease (ACVD) [82]. Here, *Streptococcus* and *Escherichia coli*, which are associated with inflammation, were enriched, while *Roseburia intestinalis* and *Faecalibacterium* cf. *prausnitzii,* which synthesize short chain fatty acids (SCFAs), were depleted in ACVD patients [82]. The Bogalusa Heart Study linked *Alloprevatella* and *Catenibacterium* enrichment with low lifetime CVD risk and *Prevotella 2*, *Prevotella 7, Tyzzerella* and *Tyzzerella 4* enrichment with high lifetime CVD risk [83], while others have shown that changes in the human microbiome are linked to CVD-related events and risks [9], including atrial fibrillation [10], heart failure [11], elevated blood lipids and cholesterol [12,13] and hypertension [84]. 

Indeed, clear differences in microbial communities and metabolites, e.g., SCFAs, were observed between normotensive and hypertensive patients [84]. Additionally, Kim et al. showed that *Ruminococcus torques, Eubacterium siraeum* and *Alistipes finegoldii* were positively associated with systolic blood pressure and intestinal inflammation, while *Bacteroides thetaiotaomicron*, which protects intestinal junctions, was negatively associated with systolic blood pressure [85]. Animal experiments have strengthened the microbiome–hypertension postulate, as germ-free mice exposed to angiotensin II-induced hypertension experienced attenuated cardiac fibrosis, inflammation and systolic dysfunction compared to their conventionally raised counterparts [86]. Finally, Adnan et al. showed an increase in the *Firmicutes:Bacteroidetes* ratio, a commonly used indicator of gut dysbiosis, and systolic blood pressure in rats gavaged microbiota from spontaneously hypertensive rodents [87]. As hypertension is one of the major risk factors for heart disease, these aforementioned studies yield promise in targeting the microbiome and particular metabolite-producing microbiota to alleviate CVD.

#### Microbiome, Histone Acetylation and CVD

Gut microbiota like *Bifidobacterium, Megasphaera massiliensis* and *Lactobacillus* synthesize short-chain fatty acid metabolites (SCFAs) from dietary fiber. SCFAs such as acetate, propionate and butyrate can enter circulation and inhibit HDAC activity in blood and select tissues including the liver, adipose tissue, brain and myocardium [14,88]. Indeed, high abundances of the SCFA-producing commensal strains, especially butyrate-producing strains, have been shown to inhibit HDACs [14]. Such effects can be protective both locally in the GI tract, as well as systemically, such as in the heart. Regarding local protection, butyrate-mediated HDAC inhibition attenuated production of pro-inflammatory cytokines by intestinal macrophages [89]. This is important as GI inflammation can result in chronic, low-grade systemic inflammation and is associated with several diseases including CVDs. Regarding systemic protection, butyrate inhibits class I HDAC activity in the heart, which attenuates cardiac hypertrophy, fibrosis and dysfunction across several experimental animal models of CVD [90,91,92]. Acetate and propionate are also HDAC inhibitors, albeit less potent [93], and have been shown to block common hallmarks of CVD upon GI microbial synthesis [94,95].

While diet-derived SCFAs are the common epigenetic link between the microbiome and heart health, evidence exists for other food stuffs in this regard. For example, mice supplemented with the probiotic *Bifidobacterium animalis* subsp. *lactis* showed attenuated ischemia/reperfusion-induced myocardial infarction and inflammation [96]. These actions were dependent upon T_reg_ cells, a type of cell important for the inflammatory response to cardiac injury [97], which showed increased histone acetylation with probiotic supplementation [96]. This is interesting as the acetylation status of T_reg_ cells was shown to regulate its anti-inflammatory function [98]. Consistent with these data, other studies have linked histone acetylation with the anti-inflammatory effects of probiotic bacterial strains [99,100]. These studies provide some foundation towards elucidating the mechanisms by which probiotics combat deleterious actions to the heart; however, further investigation is required. 

## 4. Phytochemicals and the Microbiome

Gut microbiota are involved in second-phase metabolism of phytochemicals, specifically through xenobiotic reactions of ring-cleavage, reduction, decarboxylation, demethylation and dihydroxylation reactions, which synthesize phytochemical metabolites [101]. These phytochemical metabolites are then either excreted via feces or transported into circulation as compounds that may or may not be more bioavailable and/or bioactive than their parent compound [101,102]. Thus, one interaction between phytochemicals and the microbiome that may influence host health is by synthesizing metabolites via xenobiotic metabolism. A second phytochemical–microbiome interaction that may influence host health is microbiota recomposition driven by phytochemical exposure [103,104]. Such interactions for the phytochemicals curcumin, resveratrol and EGCG are discussed below. 

### 4.1. Curcumin and the Microbiome in CVD

As the bioavailability of curcumin is low, some speculate that the gut mediates the pharmacological efficacy of curcumin. Thus far, limited research suggests that curcumin significantly affects microbial recomposition. For instance, α-diversity was not affected, while microbial abundance was affected by curcumin supplementation in mice or humans [105,106]. Specifically, the abundance of *Prevotella* genera decreased while the abundances of *Alistipes* and *Bacteroides* generas increased in mice supplemented with curcumin [105]. In humans supplemented with curcumin, *Clostridium xylanolyticum, Collinsella aerofaciens, Kluyvera intermedia and Raoultella electrica* abundances increased, while *Coprococcus catus* abundance decreased [106]. What is more promising, is that the gut metabolizes curcumin to form demethylated curcumin, which is more bioavailable and bioactive compared to its parent compound [107]. Indeed, demethylated curcumin was shown to be less toxic and more efficient at reducing oxidative stress, i.e., increased glutathione and decreased reactive oxygen species, than curcumin in HT4 neuronal cells [107]. Additionally, demethylated curcumin downregulated expression of tumor necrosis factor-alpha (TNF-α)-inducible chemokines and pro-atherosclerotic inflammatory adhesion molecules as well as apoptotic pathways more so than curcumin in human microvascular endothelial cells [107]. Thus, the anti-inflammatory properties of demethylated curcumin [107] may be efficacious to the heart. Nevertheless, neither these effects of demethylated curcumin nor the actions of demethylated curcumin on histone acetylation have been directly investigated in the heart and require further study.

An additional mechanism for microbiota-mediated curcumin activity is via enhanced SCFA production in hypertensive patients [108]. Consistent with this, curcumin-treated microbiota fermented higher concentrations of SCFAs, similar to fiber-treated microbiota, than those treated with control [108]. These data are intriguing as the efficacious actions of SCFAs have been implicated in hypertension [94]. Thus, curcumin extracts may provide cardioprotection by recomposing microbiota to ones which synthesize non-curcumin, global HDAC inhibitor compounds [14]. Consistent with this postulate, curcumin, used as a cotherapeutic, improved colon cell function via HDAC inhibition [109]. Future examination of curcumin–microbiome–histone acetylation interactions is likely to yield interesting findings for human health and disease, particularly in the regulation of CVD. In addition, it would be interesting to determine if fecal transplantation from curcumin-exposed microbiota would attenuate CVD in mammals.

### 4.2. Resveratrol and the Microbiome in CVD

Studies suggest that resveratrol can attenuate CVD by altering the composition of gut microbiota and metabolites implicated in the heart. For example, Chen et al. showed that resveratrol attenuated trimethylamine N-oxide (TMAO)-induced atherosclerosis by inhibiting microbiota-dependent trimethylamine (TMA) synthesis and inducing bile acid synthesis in mice [110]. Resveratrol also upregulated bile acid synthesis in the liver, which is critical for cholesterol catabolism and cardiac function [110,111]. Finally, resveratrol remodeled the gut microbiota and improved the *Firmicutes:Bacteroidetes* (F:B) ratio, suggesting disease-induced dysbiosis was reversed [110]. These data are in agreement with studies using other experimental models of CVD, which link the efficacious actions of resveratrol to the microbiome [112,113].

To our knowledge, only one study to date has linked the epigenetic actions of resveratrol with the gut [114]. Here, researchers linked increased sirtuin 1 expression with reduced pro-inflammatory cytokines in resveratrol-treated mice with inflamed colons [114]. Additionally, curcumin attenuated colon inflammation in this study but increased sirtuin expression to a lesser extent [114]. Of further interest, reseveratrol was shown to increase SCFA-producing microbiota in metabolically compromised men [115]. As SCFA HDAC inhibitors have been shown to attenuate cardiac remodeling and improve cardiac function [14], these data suggest that resveratrol protects the heart by a microbiome-epigenome-dependent mechanism. However, resveratrol did not affect any clinical endpoint in men with metabolic syndrome, although it should be noted that the study length may have been too short to see any improvements [115].

### 4.3. EGCG and the Microbiome in CVD

Several reports link the anti-obesogenic actions of EGCG with the microbiome [116,117,118,119,120,121]. As obesity is considered a major risk factor for CVD, these anti-obesogenic actions may indirectly protect the heart. Moreover, EGCG has been shown to improve bile acid regulation, important for proper cardiovascular function [111], high-fat-diet-induced liver damage [119,121] and gut inflammation [120]. Additionally, microbiota metabolized EGCG into compounds that reduced oxidative stress [122], which can provide further cardioprotection [18]. However, obesity has been shown to reduce bioavailability and increase excretion of EGCG [123,124,125], which may be due to obesity-induced changes in microbiota that metabolize EGCG for reabsorption [123]. Thus, EGCG may prevent obesity more so than reverse obesity. To clarify these discrepancies, prospective cohort studies with EGCG supplementation periods longer than 6 weeks in obese and non-obese individuals that employ microbiome, bioavailability and clinically relevant biomarker analyses, e.g., oxidative stress, inflammation and metabolic dysfunction, would be beneficial.

Few studies have examined the role of EGCG–microbiome–epigenome interactions in health and disease. One report showed that EGCG protected mice from high–fat–diet-induced metabolic dysfunction, which was linked to increased DNA methyltransferase 1 (DNMT1) expression and subsequent CpG hypomethylation in the colon [120]. While histone acetylation was not examined in this study [120], it should be noted that DNA methylation recruits zinc-dependent HDACs that are considered necessary for chromatin condensation and gene repression of hypermethylated DNA regions [126]. Thus, HDACs may have been involved in the epigenetic modifications observed in the colon [120]. Consistent with this, EGCG inhibited HDAC activity and reduced CpG hypermethylation in colon cancer cells [127]. The acetyl-histone-mediated actions of EGCG in the gut and whether these actions affect the heart directly or indirectly remain unclear.

## 5. Conclusions

**Figure 1:**
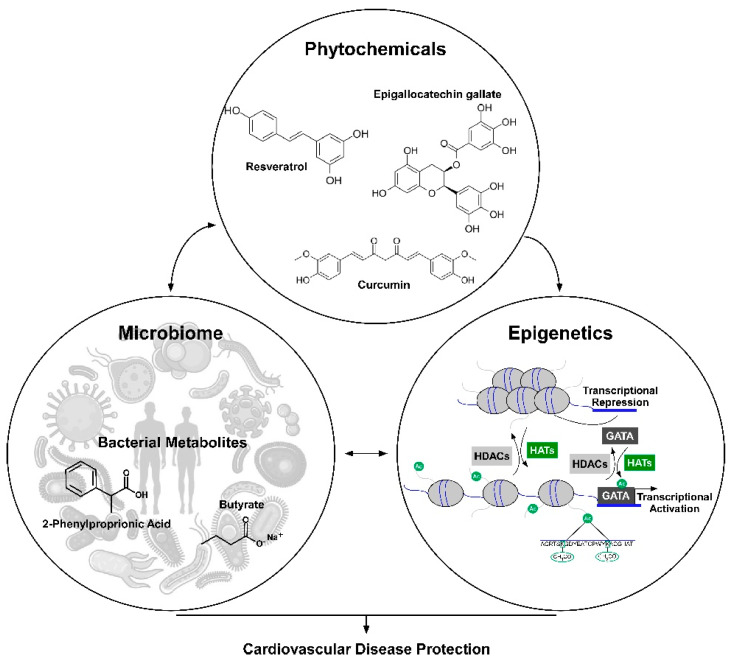
Model depicting the role for phytochemicals in the gut microbiota-histone acetylation axis in the regulation of cardiovascular disease protection. The phytochemicals curcumin, epigallocatechin gallate (EGCG) or resveratrol can alter the gut microbiome; this can promote healthy bacterial growth and bacterial metabolites needed for histone acetylation changes in the heart. The parent phytochemicals curcumin, EGCG, or resveratrol can directly regulate histone acetylation. The parent compounds or metabolites can alter histone acetylation or transcription factor activity by regulating enzymes responsible for the addition or removal of acetyl marks on lysine residues.

In this review, we characterized the cardioprotective actions of select phytochemicals. In particular, we discussed the role of curcumin, resveratrol and EGCG in the regulation of histone acetylation and the microbiome as potential therapeutic targets for CVD (Figure 1). In addition, we present findings that the gut microbiota can metabolize phytochemicals into bioactive metabolites that can increase bioavailability and improve CVD. Lastly, we review evidence that phytochemicals can alter the host microbiome, for example by changing the F:B ratio, to improve gut inflammation and host health. While many recent studies have shown that phytochemicals can attenuate or protect against CVD via HDACs/histone acetylation regulation, only a few studies have recently begun examining the impact for phytochemicals on gut microbial health and CVD. Moreover, limited if any evidence directly links the cardioprotective actions of phytochemicals with microbiome-mediated changes in histone acetylation. Thus, the examination of phytochemical–microbiome–epigenome interactions is likely to yield new and interesting future findings. It’s also important to note that we only discussed three phytochemicals in this review, but other phytochemicals have been studied in regard to heart–HDAC–histone acetylation. To this end, further examination of additional phytochemicals in microbiome–HDAC interactions will likely further our understanding of the role of diet and these food bioactives in health and CVD.

Lastly, in this review we begin to discuss the translational findings for curcumin, resveratrol and EGCG in CVD prevention and treatment. While cell and animal experiments have demonstrated a role for these three phytochemicals in the prevention and treatment of CVDs, human studies have been less clear. These discrepencies are likely due to differences in dosing strategies (e.g., oral vs. intraperitoneal delivery or every day vs. everyother day dosing), parent compound bioavailbility, synergistic/antagonistic actions with current therapeutics, as well as loss of protective microbes due to disease status and current drug treatments. Studies that examine these confounding factors will likely help clear the confusion regarding the benefits of these phytochemicals in cardiovascular diseases and may open the door for the use of these nutraceuticals as co-therapeutics.

## Abbreviations

CVD	Cardiovascular disease
HDAC	Histone deacetylase
HAT	Histone acetyltransferase
SCFA	Short-chain fatty acid
TMA/O	Trimethylamine/-N-oxide
EGCG	Epigallocatechin gallate
DNMT	DNA methyltransferase
F:B	*Firmicutes:Bacteroidetes*
TIMP1	Tissue inhibitor of metalloproteinase 1
MMP	Matrix metalloproteinase
NF-κB	Nuclear factor-kappa B
MAPK	Mitogen-activated protein kinase
GI	Gastrointestinal
ACVD	Atherosclerotic cardiovascular disease
SERCA2a	Sarcoplasmic reticulum Ca-ATPase 2a
eNOS	Endothelial nitric oxide synthase
NO	Nitric oxide
cTnI	Cardiac troponin I
ApoB	Apolipoprotein B
LDL	Low-density lipoprotein
LDLox	Oxidized low-density lipoprotein
TG	Triglycerides
TC	Total cholesterol
PPARγ	Peroxisome proliferator-activated receptor-gamma

## References

[1] Benjamin E.J., Virani S.S., Callaway C.W., Chang A.R., Cheng S., Chiuve S.E., Cushman M., Delling F.N., Deo R., de Ferranti S.D. (2018). Heart Disease and Stroke Statistics—2018 Update: A Report From the American Heart Association. Circulation.

[2] Kouzarides T. (2007). Chromatin Modifications and Their Function. Cell.

[3] Yang X.-J.J., Seto E. (2007). HATs and HDACs: From structure, function and regulation to novel strategies for therapy and prevention. Oncogene.

[4] Antos C.L., McKinsey T.A., Dreitz M., Hollingsworth L.M., Zhang C.L., Schreiber K., Rindt H., Gorczynski R.J., Olson E.N. (2003). Dose-dependent blockade to cardiomyocyte hypertrophy by histone deacetylase inhibitors. J. Biol. Chem..

[5] Williams S.M., Golden-Mason L., Ferguson B.S., Schuetze K.B., Cavasin M.A., Demos-Davies K., Yeager M.E., Stenmark K.R., McKinsey T.A. (2014). Class I HDACs regulate angiotensin II-dependent cardiac fibrosis via fibroblasts and circulating fibrocytes. J. Mol. Cell. Cardiol..

[6] Demos-Davies K.M., Ferguson B.S., Cavasin M.A., Mahaffey J.H., Williams S.M., Spiltoir J.I., Schuetze K.B., Horn T.R., Chen B., Ferrara C. (2014). HDAC6 contributes to pathological responses of heart and skeletal muscle to chronic angiotensin-II signaling. AJP Heart Circ. Physiol..

[7] Cencioni C., Spallotta F., Mai A., Martelli F., Farsetti A., Zeiher A.M., Gaetano C. (2015). Sirtuin function in aging heart and vessels. J. Mol. Cell. Cardiol..

[8] Huttenhower C., Gevers D., Knight R., Abubucker S., Badger J.H., Chinwalla A.T., Creasy H.H., Earl A.M., Fitzgerald M.G., Fulton R.S. (2012). Structure, function and diversity of the healthy human microbiome. Nature.

[9] Haghikia A., Li X.S., Liman T.G., Bledau N., Schmidt D., Zimmermann F., Kränkel N., Widera C., Sonnenschein K., Haghikia A. (2018). Gut microbiota-dependent trimethylamine N-oxide predicts risk of cardiovascular events in patients with stroke and is related to proinflammatory monocytes. Arterioscler. Thromb. Vasc. Biol..

[10] Zuo K., Li J., Wang P., Liu Y., Liu Z., Yin X., Liu X., Yang X. (2019). Duration of Persistent Atrial Fibrillation Is Associated with Alterations in Human Gut Microbiota and Metabolic Phenotypes. mSystems.

[11] Hayashi T., Yamashita T., Watanabe H., Kami K., Yoshida N., Tabata T., Emoto T., Sasaki N., Mizoguchi T., Irino Y. (2019). Gut microbiome and plasma microbiome-related metabolites in patients with decompensated and compensated heart failure. Circ. J..

[12] Fu J., Bonder M.-J., Cenit M.C., Tigchelaar E.F., Maatman A., Dekens J.A., Brandsma E., Marczynska J., Imhann F., Weersma R.K. (2015). The Gut Microbiome Contributes to a Substantial Proportion of the Variation in Blood Lipids. Circ. Res..

[13] Jones M.L., Martoni C.J., Parent M., Prakash S. (2011). Cholesterol-lowering efficacy of a microencapsulated bile salt hydrolase-activeLactobacillus reuteriNCIMB 30242 yoghurt formulation in hypercholesterolaemic adults. Br. J. Nutr..

[14] Yuille S., Reichardt N., Panda S., Dunbar H., Mulder I. (2018). Human gut bacteria as potent class I histone deacetylase inhibitors in vitro through production of butyric acid and valeric acid. PLoS ONE.

[15] Afshin A., Sur P.J., Fay K.A., Cornaby L., Ferrara G., Salama J.S., Mullany E.C., Abate K.H., Abbafati C., Abebe Z. (2019). Health effects of dietary risks in 195 countries, 1990–2017: A systematic analysis for the Global Burden of Disease Study 2017. Lancet.

[16] Chiuve S.E., Fung T.T., Rimm E.B., Hu F.B., McCullough M.L., Wang M., Stampfer M.J., Willett W.C. (2012). Alternative dietary indices both strongly predict risk of chronic disease. J. Nutr..

[17] Levitan E.B., Lewis C.E., Tinker L.F., Eaton C.B., Ahmed A., Manson J.E., Snetselaar L.G., Martin L.W., Trevisan M., Howard B.V. (2013). Mediterranean and DASH diet scores and mortality in women with heart failure: The Women’s Health Initiative. Circ. Heart Fail..

[18] Pop M., Popolo A., Trifa A.P., Stanciu L.A. (2018). Phytochemicals in Cardiovascular and Respiratory Diseases: Evidence in Oxidative Stress and Inflammation. Oxidative Med. Cell. Longev..

[19] Kurmukov A.G. (2012). Phytochemistry of Medicinal Plants. Medicinal Plants of Central Asia: Uzbekistan and Kyrgyzstan.

[20] Scalbert A., Andres-Lacueva C., Arita M., Kroon P.A., Manach C., Urpi M., Wishart D.S. (2011). Databases on Food Phytochemicals and Their Health-Promoting Effects. J. Agric. Food Chem..

[21] Vasanthi A.H.R., Shrishrimal N., Das D. (2012). Phytochemicals from plants to combat cardiovascular disease. Curr. Med. Chem..

[22] Rajkumari S., Sanatombi K. (2017). Nutritional value, phytochemical composition, and biological activities of edibleCurcumaspecies: A review. Int. J. Food Prop..

[23] Chunlai Z., Zhong P., Zhao Y., Kanchana K., Zhang Y., Khan Z.A., Chakrabarti S., Wu L., Wang J., Liang G. (2015). Curcumin protects hearts from FFA-induced injury by activating Nrf2 and inactivating NF-κB both in vitro and in vivo. J. Mol. Cell. Cardiol..

[24] Chuengsamarn S., Rattanamongkolgul S., Phonrat B., Tungtrongchitr R., Jirawatnotai S. (2014). Reduction of atherogenic risk in patients with type 2 diabetes by curcuminoid extract: A randomized controlled trial. J. Nutr. Biochem..

[25] Morimoto T., Sunagawa Y., Kawamura T., Takaya T., Wada H., Nagasawa A., Komeda M., Fujita M., Shimatsu A., Kita T. (2008). The dietary compound curcumin inhibits p300 histone acetyltransferase activity and prevents heart failure in rats. J. Clin. Investig..

[26] Yanazume T., Hasegawa K., Morimoto T., Kawamura T., Wada H., Matsumori A., Kawase Y., Hirai M., Kita T. (2003). Cardiac p300 Is Involved in Myocyte Growth with Decompensated Heart Failure. Mol. Cell. Biol..

[27] Yuan Z., Syed M.A., Panchal D., Rogers D., Joo M., Sadikot R.T. (2012). Curcumin mediated epigenetic modulation inhibits TREM-1 expression in response to lipopolysaccharide. Int. J. Biochem. Cell Biol..

[28] Kouassi K.T., Gunasekar P., Agrawal D., Jadhav G. (2018). TREM-1; Is It a Pivotal Target for Cardiovascular Diseases?. J. Cardiovasc. Dev. Dis..

[29] Liu H.-L., Chen Y., Cui G.-H., Zhou J.-F. (2005). Curcumin, a potent anti-tumor reagent, is a novel histone deacetylase inhibitor regulating B-NHL cell line Raji proliferation. Acta Pharmacol. Sin..

[30] Hu J., Shen T., Xie J., Wang S., He Y., Zhu F. (2017). Curcumin modulates covalent histone modification and TIMP1 gene activation to protect against vascular injury in a hypertension rat model. Exp. Ther. Med..

[31] Bergman M.R., Teerlink J.R., Mahimkar R., Li L., Zhu B.-Q., Nguyen A., Dahi S., Karliner J.S., Lovett D.H. (2007). Cardiac matrix metalloproteinase-2 expression independently induces marked ventricular remodeling and systolic dysfunction. Am. J. Physiol. Circ. Physiol..

[32] Kinoshita T., Ishikawa Y., Arita M., Akishima-Fukasawa Y., Fujita K., Inomata N., Suzuki T., Namiki A., Mikami T., Ikeda T. (2014). Antifibrotic response of cardiac fibroblasts in hypertensive hearts through enhanced TIMP-1 expression by basic fibroblast growth factor. Cardiovasc. Pathol..

[33] Ma J., Ma S.Y., Ding C.H. (2016). Curcumin reduces cardiac fibrosis by inhibiting myofibroblast differentiation and decreasing transforming growth factor β1 and matrix metalloproteinase 9/tissue inhibitor of metalloproteinase 1. Chin. J. Integr. Med..

[34] Ramírez-Boscá A., Soler A., Carrión M.A., Díaz-Alperi J., Bernd A., Quintanilla C., Almagro E.Q., Miquel J. (2000). An hydroalcoholic extract of Curcuma longa lowers the apo B/apo A ratio. Implications for atherogenesis prevention. Mech. Ageing Dev..

[35] Morimoto T., Funamoto M., Sunagawa Y., Katanasaka Y., Miyazaki Y., Imaizumi A., Kakeya H., Yamakage H., Satoh-Asahara N., Komiyama M. (2016). Highly absorptive curcumin reduces serum atherosclerotic low-density lipoprotein levels in patients with mild COPD. Int. J. Chronic Obstr. Pulm. Dis..

[36] Panahi Y., Khalili N., Sahebi E., Namazi S., Reiner Ž., Majeed M., Sahebkar A., Sahbekar A. (2017). Curcuminoids modify lipid profile in type 2 diabetes mellitus: A randomized controlled trial. Complement. Ther. Med..

[37] Wongcharoen W., Jai-Aue S., Phrommintikul A., Nawarawong W., Woragidpoonpol S., Tepsuwan T., Sukonthasarn A., Apaijai N., Chattipakorn N. (2012). Effects of Curcuminoids on Frequency of Acute Myocardial Infarction After Coronary Artery Bypass Grafting. Am. J. Cardiol..

[38] Sukardi R., Sastroasmoro S., Siregar N.C., Djer M.M., Suyatna F.D., Sadikin M., Ibrahim N., Rahayuningsih S.E., Witarto A.B. (2015). The role of curcumin as an inhibitor of oxidative stress caused by ischaemia re-perfusion injury in tetralogy of Fallot patients undergoing corrective surgery. Cardiol. Young.

[39] Ferguson B.S., Harrison B.C., Jeong M.Y., Reid B.G., Wempe M.F., Wagner F.F., Holson E.B., McKinsey T.A. (2013). Signal-dependent repression of DUSP5 by class I HDACs controls nuclear ERK activity and cardiomyocyte hypertrophy. Proc. Natl. Acad. Sci. USA.

[40] Chen H., Yang X., Lu K., Lu C., Zhao Y., Zheng S., Li J., Huang Z., Huang Y., Zhang Y. (2017). Inhibition of high glucose-induced inflammation and fibrosis by a novel curcumin derivative prevents renal and heart injury in diabetic mice. Toxicol. Lett..

[41] Burns J., Yokota T., Ashihara H., Lean M.E.J., Crozier A. (2002). Plant Foods and Herbal Sources of Resveratrol. J. Agric. Food Chem..

[42] Bindu S., Pillai V.B., Gupta M.P. (2016). Role of Sirtuins in Regulating Pathophysiology of the Heart. Trends Endocrinol. Metab..

[43] Sulaiman M., Matta M.J., Sunderesan N.R., Gupta M.P., Periasamy M. (2009). Resveratrol, an activator of SIRT1, upregulates sarcoplasmic calcium ATPase and improves cardiac function in diabetic cardiomyopathy. Am. J. Physiol. Heart Circ. Physiol..

[44] Sin T.K., Yu A.P., Yung B.Y.-M., Yip S.P., Chan L.W.C., Wong S.C.C., Ying M., Rudd J.A., Siu P.M. (2014). Modulating effect of SIRT1 activation induced by resveratrol on Foxo1-associated apoptotic signalling in senescent heart. J. Physiol..

[45] Van Empel V.P., Bertrand A.T., Hofstra L., Crijns H.J., Doevendans P., De Windt L.J. (2005). Myocyte apoptosis in heart failure. Cardiovasc. Res..

[46] Chen C.-J., Yu W., Fu Y.-C., Wang X., Li J., Wang W. (2009). Resveratrol protects cardiomyocytes from hypoxia-induced apoptosis through the SIRT1–FoxO1 pathway. Biochem. Biophys. Res. Commun..

[47] Mattagajasingh I., Kim C.-S., Naqvi A., Yamamori T., Hoffman T.A., Jung S.-B., DeRicco J., Kasuno K., Irani K. (2007). SIRT1 promotes endothelium-dependent vascular relaxation by activating endothelial nitric oxide synthase. Proc. Natl. Acad. Sci. USA.

[48] Folino A., Losano G., Rastaldo R. (2013). Balance of Nitric Oxide and Reactive Oxygen Species in Myocardial Reperfusion Injury and Protection. J. Cardiovasc. Pharmacol..

[49] Li J., Zhong Z., Yuan J., Chen X., Huang Z., Wu Z. (2019). Resveratrol improves endothelial dysfunction and attenuates atherogenesis in apolipoprotein E-deficient mice. J. Nutr. Biochem..

[50] Venturelli S., Berger A., Böcker A., Busch C., Weiland T., Noor S., Leischner C., Schleicher S., Mayer M., Weiss T.S. (2013). Resveratrol as a Pan-HDAC Inhibitor Alters the Acetylation Status of Jistone Proteins in Human-Derived Hepatoblastoma Cells. PLoS ONE.

[51] Palomer X., Capdevila-Busquets E., Álvarez-Guardia D., Barroso E., Pallàs M., Camins A., Davidson M.M., Planavila A., Villarroya F., Vázquez-Carrera M. (2013). Resveratrol induces nuclear factor-κB activity in human cardiac cells. Int. J. Cardiol..

[52] Choi S.Y., Piao Z.H., Jin L., Kim J.H., Kim G.R., Ryu Y., Lin M.Q., Kim H.-S., Kee H.J., Jeong J.-O. (2016). Piceatannol Attenuates Renal Fibrosis Induced by Unilateral Ureteral Obstruction via Downregulation of Histone Deacetylase 4/5 or p38-MAPK Signaling. PLoS ONE.

[53] Ooi J., Tuano N.K., Rafehi H., Gao X.-M., Ziemann M., Du X.-J., El-Osta A. (2015). HDAC inhibition attenuates cardiac hypertrophy by acetylation and deacetylation of target genes. Epigenetics.

[54] Liu Y., Ma W., Zhang P., He S., Huang D. (2015). Effect of resveratrol on blood pressure: A meta-analysis of randomized controlled trials. Clin. Nutr..

[55] Fogacci F., Tocci G., Presta V., Fratter A., Borghi C., Cicero A.F.G. (2018). Effect of resveratrol on blood pressure: A systematic review and meta-analysis of randomized, controlled, clinical trials. Crit. Rev. Food Sci. Nutr..

[56] Hoseini A., Namazi G., Farrokhian A., Reiner Ž., Aghadavod E., Bahmani F., Asemi Z. (2019). The effects of resveratrol on metabolic status in patients with type 2 diabetes mellitus and coronary heart disease. Food Funct..

[57] Baur J., Pearson K.J., Price N.L., Jamieson H.A., Lerin C., Kalra A., Prabhu V.V., Allard J.S., López-Lluch G., Lewis K. (2006). Resveratrol improves health and survival of mice on a high-calorie diet. Nature.

[58] Tomé-Carneiro J., Gonzálvez M., Larrosa M., García-Almagro F.J., Avilés-Plaza F., Parra S., Yáñez-Gascón M.J., Ruiz-Ros J.A., Garcia-Conesa M.T., Tomás-Barberán F.A. (2012). Consumption of a grape extract supplement containing resveratrol decreases oxidized LDL and ApoB in patients undergoing primary prevention of cardiovascular disease: A triple-blind, 6-month follow-up, placebo-controlled, randomized trial. Mol. Nutr. Food Res..

[59] Dyck G.J.B., Raj P., Zieroth S., Dyck J.R.B., Ezekowitz J.A. (2019). The Effects of Resveratrol in Patients with Cardiovascular Disease and Heart Failure: A Narrative Review. Int. J. Mol. Sci..

[60] Reygaert W. (2018). Green Tea Catechins: Their Use in Treating and Preventing Infectious Diseases. BioMed Res. Int..

[61] Eng Q.Y., Thanikachalam P.V., Ramamurthy S. (2018). Molecular understanding of Epigallocatechin gallate (EGCG) in cardiovascular and metabolic diseases. J. Ethnopharmacol..

[62] Pan B., Quan J., Liu L., Xu Z., Zhu J., Huang X., Tian J. (2017). Epigallocatechin gallate reverses cTnI-low expression-induced age-related heart diastolic dysfunction through histone acetylation modification. J. Cell. Mol. Med..

[63] Pan B., Xu Z., Xu Y., Liu L., Zhu J., Wang X., Nan C., Zhang Z., Shen W., Huang X. (2016). Diastolic dysfunction and cardiac troponin I decrease in aging hearts. Arch. Biochem. Biophys..

[64] Liu L., Zhao W., Liu J., Gan Y., Liu L., Tian J. (2018). Epigallocatechin-3 gallate prevents pressure overload-induced heart failure by up-regulating SERCA2a via histone acetylation modification in mice. PLoS ONE.

[65] Kranias E.G., Hajjar R.J. (2012). Modulation of cardiac contractility by the phopholamban/SERCA2a regulatome. Circ. Res..

[66] Liu D., Perkins J.T., Hennig B. (2015). EGCG prevents PCB-126-induced endothelial cell inflammation via epigenetic modifications of NF-κB target genes in human endothelial cells. J. Nutr. Biochem..

[67] Yeboah J., Crouse J.R., Hsu F.-C., Burke G.L., Herrington D.M. (2007). Brachial Flow-Mediated Dilation Predicts Incident Cardiovascular Events in Older Adults. Circulation.

[68] Widlansky M.E., Hamburg N.M., Anter E., Holbrook M., Kahn D.F., Elliott J.G., Keaney J., Vita J.A. (2007). Acute EGCG supplementation reverses endothelial dysfunction in patients with coronary artery disease. J. Am. Coll. Nutr..

[69] Lorenz M., Rauhut F., Hofer C., Gwosc S., Müller E., Praeger D., Zimmermann B.F., Wernecke K.-D., Baumann G., Stangl K. (2017). Tea-induced improvement of endothelial function in humans: No role for epigallocatechin gallate (EGCG). Sci. Rep..

[70] Wasilewski R., Ubara E.O., Klonizakis M. (2016). Assessing the effects of a short-term green tea intervention in skin microvascular function and oxygen tension in older and younger adults. Microvasc. Res..

[71] Samavat H., Newman A.R., Wang R., Yuan J.-M., Wu A.H., Kurzer M.S. (2016). Effects of green tea catechin extract on serum lipids in postmenopausal women: A randomized, placebo-controlled clinical trial. Am. J. Clin. Nutr..

[72] Venkatakrishnan K., Chiu H.-F., Cheng J.-C., Chang Y.-H., Lu Y.-Y., Han Y.-C., Shen Y., Tsai K.-S., Wang C.K. (2018). Comparative studies on the hypolipidemic, antioxidant and hepatoprotective activities of catechin-enriched green and oolong tea in a double-blind clinical trial. Food Funct..

[73] Mereles D., Buss S.J., Hardt S.E., Hunstein W., Katus H.A. (2010). Effects of the main green tea polyphenol epigallocatechin-3-gallate on cardiac involvement in patients with AL amyloidosis. Clin. Res. Cardiol..

[74] Kristen A.V., Lehrke S., Buss S., Mereles D., Steen H., Ehlermann P., Hardt S., Giannitsis E., Schreiner R., Haberkorn U. (2012). Green tea halts progression of cardiac transthyretin amyloidosis: An observational report. Clin. Res. Cardiol..

[75] Donaldson G.P., Lee S.M., Mazmanian S.K. (2015). Gut biogeography of the bacterial microbiota. Nat. Rev. Genet..

[76] Qin J., Li R., Raes J., Arumugam M., Burgdorf K., Manichanh C., Nielsen T.S., Pons N., Levenez F., Yamada T. (2010). A human gut microbial gene catalogue established by metagenomic sequencing. Nature.

[77] Koliada A., Syzenko G., Moseiko V., Budovska L., Puchkov K., Perederiy V., Gavalko Y., Dorofeyev A.E., Romanenko M., Tkach S.M. (2017). Association between body mass index and Firmicutes/Bacteroidetes ratio in an adult Ukrainian population. BMC Microbiol..

[78] Odamaki T., Kato K., Sugahara H., Hashikura N., Takahashi S., Xiao J.-Z., Abe F., Osawa R. (2016). Age-related changes in gut microbiota composition from newborn to centenarian: A cross-sectional study. BMC Microbiol..

[79] Davenport E., Mizrahi-Man O., Michelini K., Barreiro L.B., Ober C., Gilad Y. (2014). Seasonal Variation in Human Gut Microbiome Composition. PLoS ONE.

[80] Rashid M.U., Zaura E., Crielaard W., Buijs M.J., Keijser B.J.F., Nord C.E., Weintraub A. (2015). Determining the Long-term Effect of Antibiotic Administration on the Human Normal Intestinal Microbiota Using Culture and Pyrosequencing Methods. Clin. Infect. Dis..

[81] David L.A., Maurice C.F., Carmody R.N., Gootenberg D., Button J.E., Wolfe B.E., Ling A.V., Devlin A.S., Varma Y., Fischbach M.A. (2013). Diet rapidly and reproducibly alters the human gut microbiome. Nature.

[82] Jie Z., Xia H., Zhong S.-L., Feng Q., Li S., Liang S., Zhong H., Liu Z., Gao Y., Zhao H. (2017). The gut microbiome in atherosclerotic cardiovascular disease. Nat. Commun..

[83] Kelly T.N., Bazzano L.A., Ajami N.J., He H., Zhao J., Petrosino J.F., Correa A., He J. (2016). Gut Microbiome Associates With Lifetime Cardiovascular Disease Risk Profile Among Bogalusa Heart Study Participants. Circ. Res..

[84] Yan Q., Gu Y., Li X., Yang W., Jia L., Chen C., Han X., Huang Y., Zhao L., Li P. (2017). Alterations of the Gut Microbiome in Hypertension. Front. Microbiol..

[85] Kim S., Goel R., Kumar A., Qi Y., Lobaton G., Hosaka K., Mohammed M., Handberg E., Richards E.M., Pepine C.J. (2018). Imbalance of gut microbiome and intestinal epithelial barrier dysfunction in patients with high blood pressure. Clin. Sci..

[86] Karbach S.H., Schönfelder T., Brandão I., Wilms E., Hörmann N., Jäckel S., Schüler R., Finger S., Knorr M., Lagrange J. (2016). Gut Microbiota Promote Angiotensin II–Induced Arterial Hypertension and Vascular Dysfunction. J. Am. Heart Assoc..

[87] Adnan S., Nelson J.W., Ajami N.J., Venna V.R., Petrosino J.F., Bryan R.M., Durgan D. (2016). Alterations in the gut microbiota can elicit hypertension in rats. Physiol. Genom..

[88] Chriett S., Dąbek A., Wojtala M., Vidal H., Balcerczyk A., Pirola L. (2019). Prominent action of butyrate over β-hydroxybutyrate as histone deacetylase inhibitor, transcriptional modulator and anti-inflammatory molecule. Sci. Rep..

[89] Chang P.V., Hao L., Offermanns S., Medzhitov R. (2014). The microbial metabolite butyrate regulates intestinal macrophage function via histone deacetylase inhibition. Proc. Natl. Acad. Sci. USA.

[90] Patel B.M. (2017). Sodium Butyrate Controls Cardiac Hypertrophy in Experimental Models of Rats. Cardiovasc. Toxicol..

[91] Zhang L., Du J., Yano N., Wang H., Zhao Y.T., Dubielecka P.M., Zhuang S., Chin Y.E., Qin G., Zhao T.C. (2017). Sodium Butyrate Protects-Against High Fat Diet-Induced Cardiac Dysfunction and Metabolic Disorders in Type II Diabetic Mice. J. Cell. Biochem..

[92] Chen Y., Du J., Zhao Y.T., Zhang L., Lv G., Zhuang S., Qin G., Zhao T.C. (2015). Histone deacetylase (HDAC) inhibition improves myocardial function and prevents cardiac remodeling in diabetic mice. Cardiovasc. Diabetol..

[93] Waldecker M., Kautenburger T., Daumann H., Busch C., Schrenk D. (2008). Inhibition of histone-deacetylase activity by short-chain fatty acids and some polyphenol metabolites formed in the colon. J. Nutr. Biochem..

[94] Marques F.Z., Nelson E., Chu P.-Y., Horlock D., Fiedler A., Ziemann M., Tan J., Kuruppu S., Rajapakse N., El-Osta A. (2017). High-Fiber Diet and Acetate Supplementation Change the Gut Microbiota and Prevent the Development of Hypertension and Heart Failure in Hypertensive Mice. Circulation.

[95] Matziouridou C., Marungruang N., Nguyen T.D., Nyman M., Hållenius F.F. (2016). Lingonberries reduce atherosclerosis inApoe-/-mice in association with altered gut microbiota composition and improved lipid profile. Mol. Nutr. Food Res..

[96] Danilo C., Constantopoulos E., McKee L., Chen H., Regan J., Lipovka Y., Lahtinen S., Stenman L., Nguyen T.-V., Doyle K. (2017). Bifidobacterium animalis subsp. lactis 420 mitigates the pathological impact of myocardial infarction in the mouse. Benef. Microbes.

[97] Tang T.-T., Yuan J., Zhu Z.-F., Zhang W.-C., Xiao H., Xia N., Yan W., Nie S.-F., Liu J., Zhou S.-F. (2011). Regulatory T cells ameliorate cardiac remodeling after myocardial infarction. Basic Res. Cardiol..

[98] Wang L., De Zoeten E.F., Greene M.I., Hancock W.W. (2009). Immunomodulatory effects of deacetylase inhibitors: Therapeutic targeting of FOXP3+ regulatory T cells. Nat. Rev. Drug Discov..

[99] Ghadimi D., Helwig U., Schrezenmeir J., Heller K.J., De Vrese M. (2012). Epigenetic imprinting by commensal probiotics inhibits the IL-23/IL-17 axis in an in vitro model of the intestinal mucosal immune system. J. Leukoc. Biol..

[100] Bhat M.I., Kumari A., Kapila S., Kapila R. (2019). Probiotic lactobacilli mediated changes in global epigenetic signatures of human intestinal epithelial cells during Escherichia coli challenge. Ann. Microbiol..

[101] Lampe J.W., Chang J.L. (2007). Interindividual differences in phytochemical metabolism and disposition. Semin. Cancer Biol..

[102] Marín L., Miguélez E.M., Villar C.J., Lombó F. (2015). Bioavailability of Dietary Polyphenols and Gut Microbiota Metabolism: Antimicrobial Properties. BioMed Res. Int..

[103] Gomes A., Oudot C., Macià A., Foito A., Carregosa D., Stewart D., De Wiele V., Berry D., Motilva M.J., Brenner C. (2019). Berry-Enriched Diet in Salt-Sensitive Hypertensive Rats: Metabolic Fate of (Poly)Phenols and the Role of Gut Microbiota. Nutrients.

[104] Vetrani C., Maukonen J., Bozzetto L., Della Pepa G., Vitale M., Costabile G., Riccardi G., Rivellese A.A., Saarela M., Annuzzi G. (2020). Diets naturally rich in polyphenols and/or long-chain n-3 polyunsaturated fatty acids differently affect microbiota composition in high-cardiometabolic-risk individuals. Acta Diabetol..

[105] Shen L., Liu L., Ji H.-F. (2017). Regulative effects of curcumin spice administration on gut microbiota and its pharmacological implications. Food Nutr. Res..

[106] Peterson C.T., Vaughn A.R., Sharma V., Chopra D., Mills P.J., Peterson S.N., Sivamani R.K. (2018). Effects of Turmeric and Curcumin Dietary Supplementation on Human Gut Microbiota: A Double-Blind, Randomized, Placebo-Controlled Pilot Study. J. Evid.-Based Integr. Med..

[107] Khanna S., Park H.-A., Sen C.K., Golakoti T., Sengupta K., Venkateswarlu S., Roy S. (2009). Neuroprotective and Antiinflammatory Properties of a Novel Demethylated Curcuminoid. Antioxid. Redox Signal..

[108] Vamanu E., Gatea F., Sârbu I., Pelinescu D. (2019). An In Vitro Study of the Influence of Curcuma longa Extracts on the Microbiota Modulation Process, In Patients with Hypertension. Pharmaceutics.

[109] Dent P., Booth L., Roberts J.L., Poklepovic A., Hancock J.F. (2020). (Curcumin+sildenafil) enhances the efficacy of 5FU and anti-PD1 therapies in vivo. J. Cell. Physiol..

[110] Chen M.-L., Yi L., Zhang Y., Zhou X., Ran L., Yang J., Zhu J.-D., Zhang Q.-Y., Mi M.-T. (2016). Resveratrol Attenuates Trimethylamine-*N*-Oxide (TMAO)-Induced Atherosclerosis by Regulating TMAO Synthesis and Bile Acid Metabolism via Remodeling of the Gut Microbiota. mBio.

[111] Khurana S., Raufman J.-P., Pallone T.L. (2011). Bile Acids Regulate Cardiovascular Function. Clin. Transl. Sci..

[112] Qiao Y., Sun J., Xia S., Tang X., Shi Y., Luo T. (2014). Effects of resveratrol on gut microbiota and fat storage in a mouse model with high-fat-induced obesity. Food Funct..

[113] Sung M.M., Byrne N.J., Robertson I.M., Kim T.T., Samokhvalov V., Levasseur J., Soltys C.-L., Fung D., Tyreman N., Denou E. (2017). Resveratrol improves exercise performance and skeletal muscle oxidative capacity in heart failure. Am. J. Physiol. Heart Circ. Physiol..

[114] Zhang L., Xue H., Zhao G., Qiao C., Sun X., Pang C., Zhang D. (2019). Curcumin and resveratrol suppress dextran sulfate sodium-induced colitis in mice. Mol. Med. Rep..

[115] Walker J.M., Eckardt P., Aleman J.O., da Rosa J.C., Liang Y., Iizumi T., Etheve S., Blaser M., Breslow J.L., Holt P.R. (2019). The effects of trans-resveratrol on insulin resistance, inflammation, and microbiota in men with the metabolic syndrome: A pilot randomized, placebo controlled clinical trial. J. Clin. Transl. Res..

[116] Zhu J., Cai R., Tan Y., Wu X., Wen Q., Liu Z., Ouyang S.-H., Yin Z., Yang H. (2020). Preventive consumption of green tea modifies the gut microbiota and provides persistent protection from high-fat diet-induced obesity. J. Funct. Foods.

[117] Seo D.B., Jeong H.W., Cho D., Lee B.J., Lee J.H., Choi J.Y., Bae I.-H., Lee S.-J. (2015). Fermented Green Tea Extract Alleviates Obesity and Related Complications and Alters Gut Microbiota Composition in Diet-Induced Obese Mice. J. Med. Food.

[118] Henning S.M., Yang J., Hsu M., Lee R.-P., Grojean E.M., Ly A., Tseng C.-H., Heber D., Li Z. (2017). Decaffeinated green and black tea polyphenols decrease weight gain and alter microbiome populations and function in diet-induced obese mice. Eur. J. Nutr..

[119] Ushiroda C., Naito Y., Takagi T., Uchiyama K., Mizushima K., Higashimura Y., Yasukawa Z., Okubo T., Inoue R., Honda A. (2019). Green tea polyphenol (epigallocatechin-3-gallate) improves gut dysbiosis and serum bile acids dysregulation in high-fat diet-fed mice. J. Clin. Biochem. Nutr..

[120] Remely M., Ferk F., Sterneder S., Setayesh T., Roth S., Kepcija T., Noorizadeh R., Rebhan I., Greunz M., Beckmann J. (2017). EGCG Prevents High Fat Diet-Induced Changes in Gut Microbiota, Decreases of DNA Strand Breaks, and Changes in Expression and DNA Methylation ofDnmt1andMLH1in C57BL/6J Male Mice. Oxidative Med. Cell. Longev..

[121] Sheng L., Jena P.K., Liu H.-X., Hu Y., Nagar N., Bronner D.N., Settles M.L., Bäumler A.J., Wan Y.-J.Y. (2018). Obesity treatment by epigallocatechin-3-gallate−regulated bile acid signaling and its enriched Akkermansia muciniphila. FASEB J..

[122] Zhang S., Zhao Y., Ohland C., Jobin C., Sang S. (2018). Microbiota facilitates the formation of the aminated metabolite of green tea polyphenol (-)-epigallocatechin-3-gallate which trap deleterious reactive endogenous metabolites. Free Radic. Biol. Med..

[123] Chen B., Zhou J., Meng Q., Zhang Y., Zhang S., Zhang L. (2018). Comparative analysis of fecal phenolic content between normal and obese rats after oral administration of tea polyphenols. Food Funct..

[124] Fang C., Kim H., Barnes R.C., Talcott S.T., Mertens-Talcott S.U. (2018). Obesity-Associated Diseases Biomarkers Are Differently Modulated in Lean and Obese Individuals and Inversely Correlated to Plasma Polyphenolic Metabolites After 6 Weeks of Mango (*Mangifera indica* L.) Consumption. Mol. Nutr. Food Res..

[125] Novotny J.A., Chen T.-Y., Terekhov A.I., Gebauer S.K., Baer D.J., Ho L., Pasinetti G.M., Ferruzzi M.G. (2017). The effect of obesity and repeated exposure on pharmacokinetic response to grape polyphenols in humans. Mol. Nutr. Food Res..

[126] Rountree M.R., Bachman K.E., Baylin S.B. (2000). DNMT1 binds HDAC2 and a new co-repressor, DMAP1, to form a complex at replication foci. Nat. Genet..

[127] Saldanha S.N., Kala R., Tollefsbol T.O. (2014). Molecular mechanisms for inhibition of colon cancer cells by combined epigenetic-modulating epigallocatechin gallate and sodium butyrate. Exp. Cell Res..

